# Alcohol use in Early Midlife: Findings from the Age 37 Follow-Up Assessment of the FinnTwin12 Cohort

**DOI:** 10.1007/s10519-024-10212-y

**Published:** 2025-02-08

**Authors:** Megan E. Cooke, Erin Lumpe, Mallory Stephenson, Mia Urjansson, Fazil Aliev, Teemu Palviainen, Sarah J. Brislin, Maarit Piirtola, Jill Rabinowitz, Antti Latvala, Peter B. Barr, Eero Vuoksimaa, Hermine H. M. Maes, Richard Viken, Richard J. Rose, Jaakko Kaprio, Danielle M. Dick, Sari Aaltonen, Jessica E. Salvatore

**Affiliations:** 1 Department of Psychiatry, Robert Wood Johnson Medical School, Rutgers University, New Brunswick, USA; 2 Department of Psychology, Rutgers University, New Brunswick, USA; 3 Virginia Institute for Psychiatric and Behavioral Genetics, Virginia Commonwealth University, Richmond, USA; 4 Institute for Molecular Medicine Finland, University of Helsinki, Helsinki, Finland; 5 UKK Institute for Health Promotion Research, Tampere, Finland; 6 Institute of Criminology and Legal Policy, University of Helsinki, Helsinki, Finland; 7 Department of Psychiatry and Behavioral Sciences, SUNY Downstate Health Sciences University, Brooklyn, USA; 8 Department of Human and Molecular Genetics, Virginia Commonwealth University, Richmond, USA; 9 Department of Psychiatry, Virginia Commonwealth University, Richmond, USA; 10 Massey Cancer Center, Virginia Commonwealth University, Richmond, USA; 11 Department of Psychological and Brain Sciences, Indiana University, Bloomington, USA

**Keywords:** Early Midlife, Longitudinal twin-family study, Alcohol, Cohort study, Twins, Sex differences

## Abstract

This paper provides an overview of the most recent assessment, collected in early midlife, of the FinnTwin12 cohort, a population-based study of Finnish twins born in 1983–1987. The twins were invited to complete an online survey assessing a range of variables, including physical and mental health, alcohol use and problems, other substance use, and early midlife environments (e.g., parenthood). In total, 2,085 individuals (~ 40% of the original sample) completed the survey (551 complete twin pairs, 58.7% female, 37.3% monozygotic, M_age_ = 37.2 years, SD = 1.47 years, age range = 34–39 years). Individuals who participated were more likely to be female, monozygotic, and have higher parental education and less hyperactivity/impulsivity and aggression at age 12 when compared to individuals who were invited but did not participate. Parental alcohol misuse and the twins’ alcohol use and misuse at age 14 were not related to study retention. Alcohol misuse in early midlife was positively associated with nicotine dependence, lifetime use of cannabis and other drugs, trauma exposure, and depressive symptoms, and negatively associated with physical health and having biological children. These new data expand upon the wealth of measures collected as part of previous assessments, expanding the scope of work on the etiology and correlates of alcohol misuse within a longitudinal, genetically-informed framework. In addition to these new survey measures, we are planning an in-person assessment to collect physiological measurements and conduct additional in-depth phenotyping on a subset of twins who have been more intensively studied over the years.

**Supplementary Information:**

The online version contains supplementary material available at 10.1007/s10519-024-10212-y.

## Introduction

Early midlife, defined as ages 30 to 40, is an understudied but important period in the developmental course of an individual’s alcohol use. Most longitudinal studies of alcohol use and problems focus on adolescence through young adulthood, perhaps driven by the idea that many individuals “mature out” of problematic alcohol use during the transition to adulthood (Lee and Sher 2018). Nonetheless, alcohol misuse is common in early midlife, with nearly one-third of individuals in their 30s and 40s reporting past-month binge drinking in the U.S. (Substance Abuse and Mental Health Services Administration 2018). In Finland, 35% of men and 18% of women aged 35–44 reported excessive alcohol consumption in 2020 (Finnish Institute for Health and Welfare 2022). Many individuals have established long-term drinking patterns by this age (Capaldi et al. 2015; Schulenberg et al. 2016), and yet many experience new onset of alcohol use disorder (AUD) or a persistent course (Vergés et al. 2012; Meier et al. 2013). Early midlife is also a pivotal time for AUD treatment seeking (Blanco et al. 2015; Grant et al. 2015), and individuals in remission from AUD are at particularly high risk for relapse during this period (Dawson et al. 2007). Despite the importance of this developmental period for changes in alcohol-related outcomes, the etiology of early midlife alcohol problems and the influence of alcohol misuse trajectories on health and well-being are not well-characterized (Lachman 2015; Schulenberg et al. 2016).

Understanding the antecedents and consequences of early midlife drinking requires a multilevel developmental contextual approach (Windle 2010). This framework emphasizes the dynamic interplay between individual predispositions and person-level risk factors (e.g., genetic factors, personality), environmental factors (e.g., social support, neighborhood characteristics), life events, alcohol misuse, and related emotional, behavioral, and health problems across development. Indeed, there is a substantial genetic component to alcohol-related outcomes (Verhulst et al. 2015), which reflects both alcohol-specific genetic influences and non-specific genetic influences that confer risk for a broad set of externalizing disorders (Krueger et al. 2002; Kendler et al. 2003, 2011). There is also substantial evidence for gene-environment interaction (GxE) effects on alcohol use, misuse, and problems, with environments that exert less social control and create more opportunities to drink playing a particularly important role in GxE processes (Dick et al. 2007; Harden et al. 2008; Button et al. 2010; Salvatore et al. 2014; Cooke et al. 2015; Slutske et al. 2019).

Longitudinal, deeply phenotyped studies of twins are a valuable tool to investigate the interrelationships between a wide range of factors and alcohol misuse. The classical twin design may be applied to estimate the contributions of additive genetic, shared environmental, and unique environmental factors to early midlife alcohol outcomes. Multivariate twin models can also be used to evaluate whether the same or different genetic and environmental factors explain the variation in different phenotypes (e.g., alcohol misuse and social support) or in the same phenotype measured at different time points (e.g., alcohol misuse in young adulthood and alcohol misuse in early midlife) (van Dongen et al. 2012). Additionally, the twin design can strengthen inferences about the causes and consequences of alcohol misuse. The few prior longitudinal studies of alcohol misuse through early midlife studied unrelated individuals (Merline et al. 2008; Lee et al. 2012; Meier et al. 2013; Capaldi et al. 2015; Jester et al. 2016), which, like any correlational design, is prone to confounding by between-family differences. Co-twin comparisons address the possibility of familial confounding and strengthen inferences in observational research by evaluating whether differences between twins in an exposure of interest (e.g., adolescent trauma exposure) are related to differences between twins in an outcome (e.g., alcohol misuse), effectively controlling for genetic and environmental influences that twin siblings share (Lahey and D’Onofrio 2010; Frisell et al. 2012; D’Onofrio et al. 2013). This application of the twin design offers insight into the degree to which associations are consistent with a causal effect versus confounded by familial factors, with notable implications for identifying targets for preventive intervention and delineating the consequences of alcohol misuse.

### The Current Study

FinnTwin12 (FT12) is a longitudinal, population-based study of Finnish twins born 1983–1987 and ascertained at ages 11–12 years old. Over the past 25 years, the FT12 study has generated a rich longitudinal dataset with reports from parents, teachers, and peers, as well as the twins themselves, on alcohol misuse and related outcomes. Research conducted based on the FT12 study has been extensively reviewed previously (Kaprio et al. 2002; Kaprio 2006, 2013; Rose et al. 2019), and the FT12 dataset has offered valuable insight into genetic and environmental influences on alcohol misuse from adolescence through young adulthood. The present article describes the latest wave of data collection, which followed the twins into their 30s. The goals of this paper are to provide an overview of the measures completed during the early midlife assessment, describe patterns of attrition within the sample, and report associations between early midlife alcohol use and related outcomes by sex.

## Materials and methods

### Participants

The study design and first four waves of data collection in FT12 have been described previously (Kaprio 2006, 2013). Briefly, FT12 has a two-stage sampling design. The larger, first-stage is an epidemiological investigation of five consecutive birth cohorts of Finnish children. For the epidemiological sample, all Finnish twins born 1983–1987 living with at least one biological parent were identified through Finland’s Population Information System at the Finnish Digital and Population Data Services Agency and invited to complete an initial family questionnaire when the twins were 11 to 12 years old. A total of 2,724 families (87% of those identified) returned this questionnaire, and individual surveys were mailed to both parents and twins to assess their smoking, lifestyle, and health. Parents and teachers were also asked to provide ratings of the twins’ behavior. The parents reported on the birth, infancy, and childhood of the twins. Twins in the epidemiological sample were invited to participate in follow-up surveys when they were ages 14 (92% retention), 17 (75% retention), and 20–24 (mean age = 22 years, 66% retention).

Nested within this epidemiological, population-based study is the second stage: an intensively studied sub-sample targeting 1,035 families of twins and their parents. The majority of families were randomly selected for inclusion in the intensively studied sub-sample; 28% of families were selected based on elevated parental scores on the Malmӧ-Modified Michigan Alcoholism Screening Test (MmMAST) (Kristenson and Trell 1982). In addition to the surveys administered through the epidemiological study, intensive subset parents were interviewed using a semi-structured psychiatric assessment interview, the Semi-Structured Assessment of the Genetics of Alcoholism (SSAGA) (Bucholz et al. 1994), when the twins were ages 11 to 12. At age 14, the twins were interviewed with the adolescent version of the same instrument (Bucholz et al. 1994). Teachers, parents, and classmates were also asked to rate the twins’ externalizing, internalizing, and prosocial behaviors. During the young adult follow-up assessment (20–24 years), the twins completed the SSAGA a second time and were invited to provide a DNA sample and plasma for multiple omics. Across both the epidemiological and intensive samples, detailed information is available on the twins’ alcohol and other substance use, physical health, internalizing and externalizing behaviors, and environments.

In 2022, a fifth wave of data collection was initiated (*M*_age_ = 37.2 years, *SD* = 1.47 years, age range = 34.4–39.9 years) to understand the predictors and consequences of drinking across development. Twins’ addresses were updated from the Finnish Digital and Population Data Services Agency, and all twins who were alive, living in Finland, and not part of a protected group (e.g., individuals who were incarcerated or living in a nursing home) were invited to complete an online survey (*N* = 4,928) or a paper questionnaire for Swedish-speaking participants (*N* = 256). The survey assessed a wide range of variables, including current health, alcohol use and problems, internalizing and externalizing behaviors, other substance use, and salient early midlife environments (e.g., employment, parenthood, relationship status; See Supplemental Files for a copy of the survey). As this assessment occurred at the end of the COVID-19 global public health emergency (Harris 2023), impacts related to the pandemic were also assessed. Participating twins were asked to invite their romantic partners/spouses to participate in a spousal survey (similar to twins’ survey, excluding the items related to being a twin). Informed consent (online or written) was obtained from all participants. The ethics committee of the Department of Public Health of the University of Helsinki and the Institutional Review Board of Indiana University approved the FinnTwin12 study protocol from the start of the cohort. The ethical approval of the ethics committee of the Helsinki University Central Hospital District (HUS) is the most recent and covers the most recent data collection (wave 5) (HUS/2226/2021, dated September 22, 2021). The HUS reviews the study annually, and 2023’s statement is number 4/2023, dated 1 February 2023.

## Measures

### Twin Early Midlife Measures

#### Demographic Measures

##### Education Level

Twins were asked, “What schools/degrees have you completed (you can choose several alternatives).” Response options were (1) comprehensive school (9 years of comprehensive schooling up to age 16 was mandatory), (2) vocational school or corresponding school, (3) college level or corresponding level, (4) senior high school, (5) university of applied sciences, or (6) college or university. We recoded the variables to represent the highest level of education a twin reported as follows: item (1) was renamed to compulsory education only; items (2) and (3) were grouped to create vocational secondary education; item (4) was renamed to academic secondary school; items (5) and (6) were grouped to create tertiary education. The recoded response options were (1) compulsory education only, (2) vocational secondary school, (3) academic secondary school, and (4) tertiary education.

##### Employment Status

Twins were asked, “Are you currently…” (1) working (including the entrepreneur), (2) at home (e.g., a house wife/husband, a stay-at-home mother/father), (3) a student, (4) unemployed, looking for work, (5) on parental leave or child care leave, (6) retired, (7) a family caregiver, or (8) something else (please specify).

##### Financial Situation

Twins were asked, “What is your financial situation?” Response options were (1) very good, (2) fairly good, (3) moderate, (4) fairly poor, (5) very poor.

##### Living Situation

Twins were first asked, “Are you still living with your twin?” Response options were (1) yes, or (2) no. If twins responded with no, they reported the age they stopped living with their twin. In a separate question, twins were asked, “Are you living together with…?” (1) a spouse or partner, (2) a spouse or a partner and child or children, (3) a parent or both of your parents, (4) alone, (5) alone with your child or children, (6) other (e.g., dormitory or with your siblings). Only one response for this question was allowed to be selected.

##### Parenthood

Twins were asked, “Do you have children whose biological father/mother you are?” Response options were (1) no and (2) yes. Twins were also asked “Are there children living in your household whose biological father/mother you are not?” Response options were (1) no and (2) yes.

#### Substance Use Measures

##### Alcohol Use

Twins were asked whether they had ever consumed alcohol in their lifetime. They were also administered the 10-item Alcohol Use Disorders Identification Test (AUDIT) to assess past-year alcohol consumption and problems (Saunders et al. 1993). The AUDIT had high internal consistency in our sample (α = 0.84), consistent with previous literature (Selin 2003). Responses to the AUDIT were summed to create a score indicating the severity of the twin’s alcohol use. A score of 8 or greater on this scale is considered hazardous alcohol use.

##### Nicotine Use

Twins were asked, “Over your lifetime have you smoked more than 100 cigarettes (5 packs)?” Response options were (1) no and (2) yes. Twins who responded “yes” were then asked, “Which of the following alternatives describes best your current use of cigarettes?” Response options were (1) I smoke daily, (2) I smoke once a week or more often, though not daily, (3) I smoke less frequently than once a week, and (4) I have stopped or quit smoking. Twins who reported smoking over 100 cigarettes in their lifetime were also asked the six questions from the Fagerström Test for Cigarette Dependence (FTCD) (Heatherton et al. 1991; Fagerström 2012) assessing highest lifetime cigarette use. The FTCD scale had high internal consistency in our sample (α = 0.78), exceeding most reliability estimates in prior literature (Sharma et al. 2021). A sum score was created, with higher scores representing more severe nicotine dependence. Twins were also asked, “Do you currently use nicotine-containing e-cigarettes?” Response options were (1) daily, (2) occasionally, or (3) no. Twins were asked if they smoked cigars, cigarillos, or pipes. Response options were (1) never, (2) once in a while, or (3) regularly. Lastly, twins were asked, “Have you tried snus (Swedish style snuff)? So far, how many times altogether?” Response options were (1) I have not tried, (2) I have tried once, (3) I have used snus 2 to 50 times, (4) I have used snus over 50 times, (5) I use snus regularly.

##### Lifetime Marijuana Use

Twins were asked, “Have you ever tried cannabis (hash or marijuana)?” Response options were (1) never, (2) 1–3 times, (3) 4–9 times, (4) 10–19 times, or (5) 20 or more times.

##### Lifetime Illicit Drug Use

Twins were asked about their lifetime illicit drug use with the question, “Have you ever used other substances to get high (thinner or other inhaled substance, amphetamine, medication on purpose to get high or other such substances)?” Response options were (1) not once, (2) 1–3 times, (3) 4 to 9 times, (4) 10 to 19 times, or (5) 20 times or more.

#### Physical Health Measures

##### Self-Rated Health

Twins were asked, “What do you think about your current health, is it…?” Response options were (1) very good, (2) fairly good, (3) moderate, (4) fairly poor, or (5) very poor (Silventoinen et al. 2007; Jylhä 2009).

##### Physical Fitness

Twins were asked, “Is your current physical fitness…?” Response options were (1) very good, (2) quite good, (3) satisfactory, (4) quite poor, or (5) very poor (Waller et al. 2019).

##### Recurrent Pain

Twins were asked to rate the frequency of their “stomachaches,” “headaches,” “low back pain,” and “neck or shoulder pain” during the past 6 months. Response options were (1) seldom or never, (2) approximately once a month, (3) approximately once a week, or (4) nearly every day (Aarnio et al. 1997; Mikkelsson et al. 2001; Kaartinen et al. 2019). We report the number and percentage of twins who reported daily pain in any of the four areas.

##### Sleep Difficulties

Twins were asked to rate the frequency of “difficulty getting to sleep” and “waking up during sleep” during the past 6 months. Response options were (1) seldom or never, (2) approximately once a month, (3) approximately once a week, or (4) nearly every day (Partinen and Gislason 1995; Aarnio et al. 1997).

#### Mental Health Measures

##### Life Satisfaction

Twins were administered the Satisfaction with Life Scale, a 5-item scale used to measure global cognitive judgments of one’s life satisfaction (Diener et al. 1985). The Satisfaction with Life Scale had high internal consistency (α = 0.90) in our sample, consistent with previous literature (Diener et al. 1985).Scores were summed to create a scale from 5 to 35, with a score of 20 being neutral. Scores above 20 represent higher life satisfaction; scores below 20 represent lower life satisfaction.

##### Previous Psychiatric Diagnoses

Twins were asked whether a physician, nurse, or other healthcare provider had ever told them they had the following psychiatric conditions: anxiety or panic disorder, post-traumatic stress disorder (PTSD), attention deficit hyperactivity disorder (ADHD), or depression.

##### Depressive Symptoms

Twins were administered the modified 8-item Center for Epidemiological Studies Depression Scale (CES-D), a self-report questionnaire designed to measure frequency of past-week depressive symptoms in research populations (Radloff 1977; Van de Velde et al. 2010; Briggs et al. 2018). The CES-D had high internal consistency within our sample (α = 0.83), consistent with previous estimates in the literature (Briggs et al. 2018). Twins were asked to rate their frequency of the following: (1)”I felt depressed”, (2) “I felt that everything I did was an effort”, (3) “My sleep was restless”, (4) “I was happy”, (5) “I felt lonely”, (6) “I enjoyed life”, (7) “I felt sad”, and (8) “I could not get going”. Response options were (0) rarely or none of the time (less than 1 day), (1) some or a little of the time (1–2 days), (2) occasionally or a moderate amount of the time (3–4 days), and (3) most or all of the time (5–7 days). Items assessing positive behaviors (items 4 and 6) were reverse coded. Scores were summed to create a range between 0 and 24.

##### Lifetime Traumatic Events

Twins were asked whether they had experienced the following events in their lifetime: own divorce or separation, death of someone close to them, disease or injury causing over three weeks of work disability, serious traffic accident or other accident, fire or catastrophe, hit or kicked hard enough to get injured, forced/tried to force sexual contact, and a violent crime where a gun, knife or some other weapon was used (Carlson et al. 2011). The number of events endorsed was tallied to create a total count of traumatic events experienced.

#### COVID-related Measures

##### COVID Life Events and Stressors

Twins were asked, “Overall, considering all the possible ways your life may have been impacted by the COVID-19 pandemic, how much has the pandemic impacted your day-to-day life?” Response options were (1) it has not impacted my life at all, (2) it has impacted my life a little, (3) it has moderately impacted my life, and (4) it has extremely impacted my life. If twins responded with options 2–4, they were asked to rate how the COVID-19 pandemic affected a variety of areas in their life, including communication, physical activity, work, and stress (Johns Hopkins University 2020; Finnish Institute for Health and Welfare).

### Twin Measures for Attrition Analyses

#### Biological Sex

Biological sex was ascertained from the personal data provided by Finnish Central Population Registry prior to the age 12 assessment.

#### Zygosity

Zygosity was first assessed at age 12 with a questionnaire that determined appearance similarity (Sarna et al. 1978). Zygosity was later confirmed with genetic markers.

#### Frequency of Alcohol use and Intoxication

Frequency of alcohol use and intoxication were measured at the age 14 assessment. The first question asked twins, “How often do you drink alcohol?” The second question asked, “How often do you drink so that you get at least slightly intoxicated?” Response options for both questions were (1) once a week or more, (2) about 1–2 times a month, (3) less than once a month, and (4) never; I don’t drink alcohol. Response options were reverse coded.

#### Hyperactivity/Impulsivity, Aggression and Depression

Parents reported on the twins’ emotional, behavioral, and social problems at twins’ age 12 assessment using the Multidimensional Peer Nomination Inventory (MPNI) (Pulkkinen et al. 1999). To assess hyperactivity and impulsivity, parents were asked to indicate if twins were restless, acted before thinking, talked all the time, were too impatient to wait their turn, ran and climbed everywhere despite warnings, were disobedient, and were hyperactive before school age. To assess aggression, parents were asked to rate whether the twins suggested to others not to be with them, teased other people and were violent for no reason, spread rumors about other people’s personal matters when mad at them, might hit, kick, push, or throw something when angry at someone, teased smaller/weaker children, and scolded people. To assess depression, parents were asked to indicate if twins were sad or depressed, easily hurt if other people were mean to them, were lonely and had no friends, often worried, and clung to adults or was too dependent on them. Response options for each measure were (0) not noticeable, (1) sometimes noticeable, (2) fits rather clearly, and (3) fits very well. Responses were summed, and mean scores for hyperactivity/impulsivity, aggression, and depression were calculated (Whipp et al. 2019, 2022).

### Parental Measures for Attrition Analyses

#### Alcohol Misuse

Problematic alcohol use in parents was assessed using the MmMAST at the twins’ age 12 assessment (Kristenson and Trell 1982). The MmMAST is an 11-question yes/no survey, with 9 questions that assess typical drinking habits and 2 questions that assess alcohol problems consistent with DSM-IV AUD criteria (Sipilä et al. 2023).

#### Educational Attainment

Educational attainment in both mothers and fathers was assessed during the twins’ age 12 assessment. Parents were asked, “What is your basic education?” Response options were (1) less than elementary school (less than 6 years), (2) elementary school (6–8 years), (3) less than intermediate school (4 years basic education, under 5 years of intermediate school), (4) intermediate or comprehensive school (9 years), (5) part of senior high school (under 3 years), or (6) high school diploma (12 years) (Lajunen et al. 2012).

#### Analytic Plan

We report basic descriptive statistics for the key early midlife study measures using means (*M*) and standard deviations (*SD*) for continuous variables and number and percent of respondents for categorical variables. We tested for sex differences in these measures using t-test and chi-square tests as appropriate. We created an UpSet plot (Lex et al. 2014) using UpSetR (Conway et al. 2017) to visualize the previous patterns of survey responses among those who completed the early midlife survey.

We conducted a series of attrition analyses using the pglm R package (Croissant 2021), which accounts for familial nesting, to determine if the baseline (age 12) characteristics of respondents to the early midlife survey significantly differed from non-responders. Participation at early midlife was coded as (0) = did not participate and (1) = participated. As noted above, adolescent alcohol use measures came from the twins’ age 14 assessment, as alcohol use was not measured in the age 12 assessment.

Because the early midlife data collection was funded by the National Institute on Alcohol Abuse and Alcoholism with a focus on understanding the correlates and consequences of drinking in early midlife, we ran a random effects model, using the “random” option in the model argument of the plm package (Croissant and Millo 2008), to test the association between AUDIT scores and the other sociodemographic, health, and environmental factors measured in early midlife while accounting for the nesting of individual twins within their families. We started with univariable models to examine each correlate separately with sex as a covariate. Next, we included a sex-by-correlate interaction term to test for potential sex differences. Finally, we ran a multivariable model that included all of the early midlife sociodemographic, health, and environmental factors plus sex as a covariate to examine their unique and specific associations with AUDIT scores. The threshold for statistically significant *p-*values were set to *p <*.05.

## Results

### Participation

**Fig. 1:**
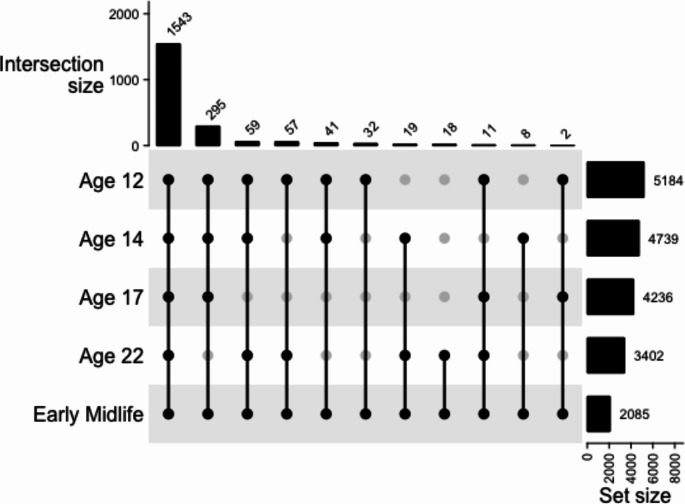
Upset plot visualizing combinations of prior survey completion for those who completed the early midlife survey (*N* = 2,085)

A total of 2,085 individuals from 1,498 families completed the early midlife survey. This represents 61.3% of the individuals who completed the young adulthood survey (*M*_age_ = 22 years) and 40.2% of the individuals who completed the baseline survey at age 12 (*N* = 5,184). Most participants who completed the early midlife survey also completed the surveys at ages 12, 14, and 17 (see Fig. 1 for response patterns of participants across the five survey administrations of the project). A total of 437 partners of the twins filled out the partner survey. The sex breakdown for the partners was 229 (52.4%) female, 207 (47.4%) male, and 1 person responding “other”. The partners ranged in age from 27 to 65 with a mean age of 38.59 (*SD* = 5.06). The partner/spouse data will be explored in a subsequent report and thus is not discussed here.

This graph represents the response patterns for all twins who completed the early midlife assessment. The rows or “set size” represent the number of twins that participated in each assessment. The columns or “intersection size” represents the number of twins that participated in the response pattern below the bar. A black dot represents which surveys were completed and therefore included in each intersection set. Twins that were invited to participate at age 12 (but may not have answered) were invited to participate in subsequent surveys.

### Attrition Analyses

Females (OR: 2.29, 95% CI: 1.95–2.72), monozygotic (MZ) twins (OR: 1.39, 95% CI: 1.15–1.69), and twins with higher parental education (maternal: OR: 1.13, 95% CI: 1.07–1.20; paternal: OR: 1.07, 95% CI: 1.01–1.14) were more likely to participate in the early midlife follow-up survey. Twins with lower hyperactivity or impulsivity (OR: 0.59, 95% CI: 0.51–0.70) and lower aggression (OR: 0.67, 95% CI: 0.54–0.83), measured at age 12, were more likely to participate at the early midlife follow-up. Parental alcohol misuse reported at the twins’ age 12 assessment (mother: OR: 1.05, 95% CI: 0.41–1.21; father: OR: 0.95, 95% CI: 0.88–1.02) and parent report of the twins’ depression at age 12 (OR: 0.92, 95% CI: 0.76–1.11) were not significantly associated with participation in the early midlife follow-up. Twin frequency of alcohol use (OR: 1.04, 95% CI: 0.95–1.15) and frequency of intoxication (OR: 1.08, 95% CI: 0.96–1.22), measured at age 14, were not significantly associated with participation in the early midlife follow-up. A table of the full attrition analyses can be found in the Supplemental Materials.

### Demographics

Of those who participated in the early midlife survey, 58.7% were female (*N* = 1,224). The zygosity breakdown of the participants was as follows: 292 male MZ twins (MZM, 14.0%, 89 complete pairs), 274 male dizygotic (DZ) twins (DZM, 13.1%, 62 complete pairs), 440 female MZ twins (MZF, 21.1%, 157 complete pairs), 326 female DZ twins (DZF, 15.6%, 96 complete pairs), and 629 opposite sex DZ twins (DZO, 30.2%, 147 complete pairs). The basic demographics for the sample along with tests of sex differences are shown in Table 1. Twins reported an average life satisfaction score of 24.92 (*SD* = 6.19), slightly above the neutral score of 20 (*M*_males_ = 24.84, *SD* = 6.16; *M*_females_ = 24.98, *SD* = 6.21; *t*(1816.1) = -0.527, *p* =.598).

**Table 1:** Early midlife sample demographics

	Male		Female		Total	
	*N*	%	*N*	%	*N*	%
MZ	292	33.9	440	35.9	732	35.1
DZ	274	31.8	326	26.6	600	28.8
DZO	254	29.5	375	30.6	629	30.2
Unknown Zygosity	41	4.8	83	6.8	124	5.9
Education**						
Compulsory education only	19	2.2	18	1.5	37	1.78
Vocational secondary	252	29.3	228	18.6	480	23.0
Academic secondary	105	12.2	126	10.3	231	11.1
Tertiary education	483	56.1	846	69.1	1329	63.7
Missing	2	0.2	6	0.5	8	0.4
*Employment*						
Working	790	91.8	992	81.0	1782	82.9
At home	2	0.2	21	1.7	23	1.1
Student	18	2.1	60	4.9	78	3.7
Unemployed, looking for work	25	2.9	22	1.8	47	2.3
On maternity, paternity, parental or child care leave	3	0.3	101	8.3	104	5.0
Retired	16	1.9	18	1.5	34	1.6
Family caregiver	2	0.2	1	0.1	3	0.1
Something else	11	1.3	16	1.3	27	1.3
Financial Situation**						
Very good	180	20.9	179	14.6	359	17.2
Fairly good	393	45.6	484	39.5	877	42.1
Moderate	240	27.9	455	37.2	695	33.3
Fairly poor	38	4.4	84	6.9	122	5.9
Very poor	8	0.9	17	1.4	25	1.2
Missing	2	0.2	5	0.4	7	0.3
Living Situation**						
Spouse or partner	253	29.4	323	26.4	576	27.6
Spouse/partner and child/children	371	43.1	575	47.0	946	45.4
Parents	12	1.4	6	0.5	18	0.9
Alone	196	22.8	193	15.8	389	18.7
Child or children	23	2.7	107	8.7	130	6.2
Other	4	0.5	15	1.2	19	0.9
Missing	2	0.2	5	0.4	7	0.3
Biological children**	480	55.7	784	64.1	1264	60.6
Non-biological children	68	7.9	82	6.7	150	7.2

Note. ** = a significant difference between males and females at *p* <.001. MZ = monozygotic twins, DZ = dizygotic twins, DZO = opposite sex dizygotic twins. Percentages for Employment status may not equal 100% because participants could select more than one option

### Substance Use

Table 2 shows the lifetime and current rates of substance use in the sample. Alcohol was the most used substance. The second and third most used substances were cigarettes and snus, respectively. The average FTCD score among current and former smokers was 2.42 (*SD* = 2.37), with an average score of 2.66 (*SD* = 2.40) among males and an average score of 2.23 among females (*SD* = 2.32, *t*(898.61) = 2.75, *p* =.006) indicating low levels of nicotine dependence in the sample. Patterns of early midlife alcohol use in the sample are detailed in Table 3. Males reported drinking more frequently, consuming a greater number of drinks in a typical day, more frequent binge drinking, and higher total AUDIT scores than females (*p* <.05). Males were also more likely to be drinking at hazardous levels compared to females, as indicated by proportion of individuals scoring > 8 on the AUDIT.

**Table 2:** Rates of lifetime and current substance use

	Male		Female		Total	
	*N*	%	*N*	%	*N*	%
*Alcohol*						
Lifetime alcohol use	807	93.7	1160	94.8	1967	94.3
*Other Substances*						
Lifetime cannabis use*	369	42.9	367	30.0	736	35.3
Lifetime other drug use*	131	15.2	133	10.9	264	12.7
*Nicotine*						
Lifetime cigarette use*	439	52.3	530	44.2	969	47.6
Lifetime cigar use*	167	19.4	43	3.5	210	10.1
Lifetime snuff use*	525	61.0	352	28.8	877	42.1
Current daily smoking	84	9.8	128	10.5	212	10.2
Current daily e-cigarette use	11	1.3	6	0.5	17	0.8

Note. * = a significant difference between males and females at *p* <.05

**Table 3:** Alcohol consumption and problems in early midlife

	Male		Female		Total	
	*N* / M	% / SD	*N* / M	% / SD	*N* / M	% / SD
*Categorical Variables*						
Frequency of alcohol use*						
Never	39	4.53	101	8.25	140	6.71
Monthly or less	229	26.60	542	44.28	771	36.98
2–4 times a month	341	39.61	411	33.58	752	36.07
2–3 times a week	172	19.98	96	7.84	268	12.85
4 or more times a week	35	4.07	17	1.39	52	2.49
Missing	45	5.23	57	4.66	102	4.89
Drinks on typical day*						
1 or 2	340	39.49	658	53.76	998	47.87
3 or 4	179	20.79	263	21.49	442	21.20
5 or 6	113	13.12	126	10.29	239	11.46
7 to 9	94	10.92	69	5.64	163	7.82
10 or more	84	9.76	27	2.21	111	5.32
Missing	51	5.92	81	6.62	132	6.33
Binge drinking*						
Never	54	6.27	223	18.22	277	13.29
Less than monthly	473	54.94	779	63.64	1252	60.05
Monthly	179	20.79	107	8.74	286	13.72
Weekly	96	11.15	42	3.43	138	6.62
Daily or almost daily	10	1.16	3	0.25	13	0.62
Missing	49	5.69	70	5.72	119	5.70
AUDIT Hazardous Use*	286	33.21	161	13.15	447	21.44
*Continuous Variables*						
AUDIT Total Score*	6.87	4.89	4.40	3.73	5.42	4.42

Note. Participants were asked to report past-year alcohol consumption and problems. N/M indicates the number of individuals (N) or mean (M) as appropriate. %/SD indicates percentage of the sample (%) or standard deviation (SD) as appropriate. * = a significant difference between males and females at *p* <.05

### Physical Health

Most twins, 69.7% (*N* = 1,453), reported being in “quite good” or “very good” health, which was similar across males (71.7%) and females (68.3%, χ^2^(4) = 6.67, *p* =.155). Relatedly, most twins, 78.2% (*N* = 1,630), reported their physical fitness was “satisfactory” or “quite good”, which was similar across males (77.1%) and females (78.1%). A subset of twins, 12.5% (*N* = 260), reported daily pain, such as stomachaches, headaches, lower back pain, neck pain, or shoulder pain. Reports of daily pain were more prevalent in females (15.5%) compared to males (8.1%, χ^2^(1) = 24.44, *p* <.001). A portion of twins, 18.4% (*N* = 383), reported having daily sleep difficulties, such as waking during sleep or difficulties getting to sleep. This occurred more frequently in females (21.4%) than in males (14.1%, χ^2^(1) = 17.70, *p* <.001).

### Mental Health

The self-reported rates of a previously diagnosed mental health condition by a health care provider were 16.8% (*N* = 350) for anxiety or panic disorder (10.2% of males, 21.4% of females, χ^2^(1) = 44.41, *p* <.001), 2.9% (*N* = 61) for PTSD (0.9% of males, 4.3% of females, χ^2^(1) = 19.43, *p* <.001), 2.2% (*N* = 45) for ADHD (1.9% of males, 2.4% of females, χ^2^(1) = 0.42, *p* =.516), and 18.9% (*N* = 393) for depression (12.0% of males, 23.7% of females, χ^2^(1) = 44.72, *p* <.001). Twins reported an average CES-D score of 5.28 (*SD* = 4.00), individual scores above 9 are suggestive of clinical depression (Radloff 1977; Briggs et al. 2018). Female twins reported higher CES-D scores (*M* = 5.59, *SD* = 4.12) than male twins (*M* = 4.85, *SD* = 3.79, *t*(1869.7) = -4.17, *p* <.001). Twins experienced an average of 1.88 traumatic events in their lifetime (*SD* = 1.39), with females (*M* = 1.98, *SD* = 1.44) reporting a greater number of traumatic events than males (*M* = 1.74, *SD* = 1.31, *t*(1885.8) = -3.88, *p* <.001).

### Experience with the COVID-19 Pandemic

**Fig. 2:**
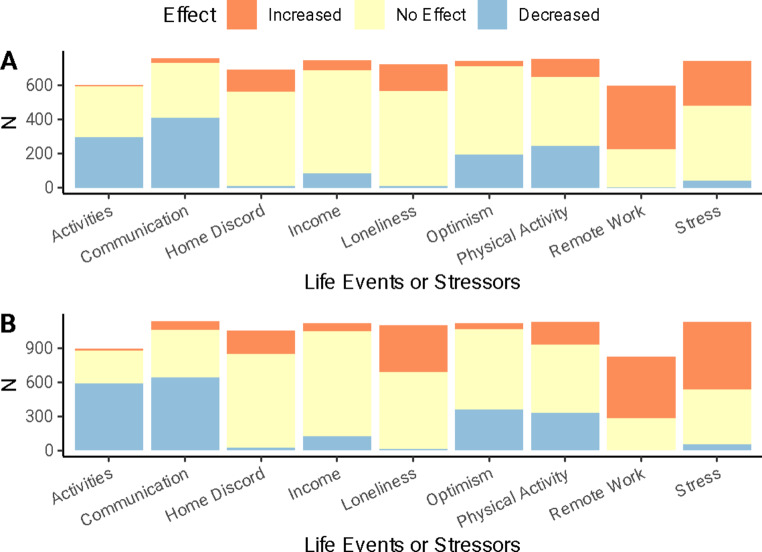
Reported effects of the COVID-19 pandemic by sex

Most twins reported that the pandemic had “impacted [their] life a little” (55.3%, *N* = 1,153, 61.0% of males, 51.4% of females). The next most common response was that the COVID-19 pandemic had “moderately impacted [their] life” (27.1%, *N* = 565, 21.1% of males, 31.3% of females). Finally, approximately the same number of twins reported that the COVID-19 pandemic had “not impacted [their] life at all” (7.2%, *N* = 150, 9.9% of males, 5.3% of females) as did those who reported that it “extremely impacted [their] life” (7.1%, *N* = 148, 5.5% of males, 8.3% of females). There were significant differences in the global impact of the pandemic based on sex, χ^2^(3) = 47.45, *p* <.001. Figure 2 displays the specific areas of life that twins reported were impacted by the COVID-19 pandemic by sex. More females reported an increase in loneliness and stress, and a decrease in optimism and time spent at school, social, and religious activities compared to males (*p*s < 0.05). In addition, female twins more commonly reported a change (either increase or decrease) in communication with friends, family members, and relatives compared to male twins (*p* =.003).

This graph shows the extent to which male and female twins reported certain life events and stressors being impacted by the COVID-19 pandemic. Panel A shows the distributions in male twins. Panel B shows the distributions in female twins.

### AUDIT Analyses

Table 4 shows the univariable and multivariable associations with AUDIT total scores in early midlife. In the univariable models, lifetime cigarette use, higher FTCD scores, lifetime cannabis use, lifetime use of other drugs, experiencing more traumatic events, and higher depression scores were significantly associated with higher AUDIT scores. In contrast, better self-reported physical health, financial situation, and having biological children were associated with lower AUDIT scores. There were significant sex-by-correlate interactions for lifetime cigarette use, depression, physical health, and financial situation, such that there was a stronger association in males compared to females. In the multivariable model, the effect size decreased for most predictors, and some were no longer significant (i.e., lifetime cigarette use, self-reported financial situation). Having biological children in early midlife was the exception. The association between having biological children and lower AUDIT scores was stronger in the multivariable model compared to the univariable model.

**Table 4:** Results of univariable and multivariable random effects models predicting AUDIT scores (*N* = 1,940)

Predictor	Univariable $\:\varvec{\beta\:}$ [95% CI]	Multivariable $\:\varvec{\beta\:}$ [95% CI]
Lifetime cigarette use#	0.55 [0.47, 0.63]	-0.66 [-1.98, 0.66]
Male	0.68 [0.53, 0.83]	N/A
Female	0.46 [0.36, 0.55]	N/A
FTCD score	0.22 [0.14, 0.29]	0.14 [0.07, 0.21]
Lifetime cannabis use	0.61 [0.53, 0.70]	0.28 [0.13, 0.42]
Lifetime use of other drugs	0.79 [0.67, 0.91]	0.29 [0.12, 0.46]
Lifetime traumatic events	0.22 [0.18, 0.26]	0.10 [0.03, 0.16]
Depression#	0.20 [0.16, 0.24]	0.20 [0.12, 0.27]
Male	0.30 [0.22, 0.38]	N/A
Female	0.14 [0.09, 0.19]	N/A
Physical health#	-0.17 [-0.21, -0.13]	-0.10 [-0.17, -0.02]
Male	-0.27 [-0.34, -0.19]	N/A
Female	-0.10 [-0.15, -0.05]	N/A
Financial situation#	-0.13 [-0.18, -0.09]	-0.05 [-0.12, 0.02]
Male	-0.26 [-0.34, -0.18]	N/A
Female	-0.05 [-0.10, -0.004]	N/A
Having biological children	-0.30 [-0.39, -0.21]	-0.33 [-0.47, -0.19]

Note. # indicates a significant sex-by-correlate interaction in the univariable model in which case separate effect sizes for males and females are presented

## Discussion

This manuscript presents an overview of participation and measures collected during early midlife (ages 30–40) in the longitudinal, population-based FT12 sample. Extending the FT12 cohort into early midlife provides an opportunity for researchers to explore an understudied developmental period using longitudinal, genetically-informative methods. Given that many of the behaviors and environmental factors in early midlife are genetically influenced (Kendler and Baker 2007; Kendler et al. 2008), a sample of twins with longitudinal data allows for a diverse set of analyses, including those that can explicitly quantify genetic influences (traditional biometrical twin models) or provide increasingly strict control of familial influences to understand the nature of the associations between behaviors such as alcohol use and its correlates (co-twin comparison models).

The early midlife data collection builds on the cohort’s already rich longitudinal data, with 40% of the original cohort of 5,184 twins completing the follow-up assessment approximately 25 years later. Those who participated in the early midlife assessment had fewer adolescent behavior problems than those who did not, mirroring prior reports from other longitudinal samples (Boys et al. 2003). Participants who completed the early midlife survey also reported higher parental education levels than those who did not. Previous longitudinal studies using non-twin samples report associations between higher alcohol use and survey non-responsiveness (Nwaru et al. 2021; Thygesen et al. 2008). In contrast, early midlife survey respondents in the FinnTwin12 sample did not significantly differ from nonrespondents on alcohol use measures (both twin and parent) at age 14. There are limited extant studies examining how alcohol use is associated with attrition in longitudinal twin studies; therefore, sample characteristics (i.e., twins vs. singletons) may contribute to these attrition differences. In line with prior literature (Marmorstein 2009), early midlife respondents did not significantly differ from nonrespondents on parent-reported depression at age 12. Overall, these findings indicate that those who participated in the early midlife follow-up were representative of the broader sample in terms of parent and adolescent alcohol use and twin adolescent depression. Future studies will be able to leverage the representativeness of longitudinal drinking patterns in FT12 to test theories of maturing out of problem alcohol use (Lee and Sher 2018).

As a point of comparison for the overall retention rate, recent national surveys have reported initial participation rates between 45 and 66% (Mindell et al. 2015) and a decrease in participation is common in longitudinal studies as age and number of follow-ups increase (Shulruf et al. 2007; Teague et al. 2018). Although the lower response rate observed here was unexpected given the past Finnish reputation for exceptionally high participation in health-related research (Eloranta and Auvinen 2015), it is worth noting that these data were collected during the public health emergency phase of the COVID-19 global pandemic, which stretched societies and health systems (Karreinen et al. 2023). Investigators of other longitudinal cohort studies in the United Kingdom and United States report similar challenges with participant retention for data collection efforts that occurred during the public health emergency (Nooner et al. 2020; Wels et al. 2022).

Generally, early midlife participants were thriving, reporting high levels of educational attainment, strong financial situation, high rates of employment, and little to no impact of the COVID-19 pandemic on their life. Almost all participants reported alcohol use; however, less than half reported nicotine or cannabis use within their lifetime. Of the participants who reported drinking alcohol in their lifetime, higher alcohol misuse (measured by total AUDIT score) was associated with more severe nicotine dependence, lifetime use of cannabis and other drugs, higher depression scores, and experiencing more traumatic events. Higher alcohol misuse was also associated with lower self-reported physical fitness and a decreased likelihood of being a parent in early midlife. Of note, lifetime cigarette use and financial situation did not remain significant in the multivariable model, suggesting that other measures of early midlife nicotine use (i.e., FTCD), demographics (i.e., parenthood), and health (i.e., depression, physical health, and substance use) are more robust correlates of early midlife alcohol misuse. These findings are discussed in the context of previous studies and rates in Finland broadly below.

A comparison of early midlife measures between males and females provided evidence of sex differences in demographic, substance use, physical, and mental well-being domains. Of note, males were more likely to report lifetime use of cannabis, nicotine (i.e., cigarette, cigar, and snuff), and other drugs compared to females. Males and females reported lifetime alcohol use at similar rates; however, males reported more alcohol consumption and problems than females on all measures of current (i.e., early midlife) alcohol use. We also observed sex differences in the strength of the relationship between problem alcohol use (measured by the AUDIT) and lifetime cigarette use, depression, physical health, and financial situation. Given the statistical differences between males and females on multiple early midlife measures, we recommend that future studies analyzing the FT12 early midlife data include explicit sex comparisons in all reported results.

Our assessment of early midlife drinking patterns in Finnish twins is comparable to midlife drinking trends in Finland (Tigerstedt et al. 2020b). While drinking within Finland has decreased since the beginning of the century, rates of alcohol use and problems remain high, particularly among middle-aged and older adults (Tigerstedt et al. 2020b; Warpenius and Mäkelä 2020). According to the Finnish Institute for Health and Welfare (2022), 35% of men and 18% of women aged 35–44 reported excessive alcohol consumption in 2020, only slightly higher than the 33% of males and 13% of females who reported hazardous alcohol use in our early midlife twin sample. On trend with sex differences in global drinking patterns (Bloomfield et al. 2001; Kuntsche et al. 2011; Virtanen et al. 2019; Tigerstedt et al. 2020a), female alcohol use has increased in Finland during the past decades (Finnish Institute for Health and Welfare 2022). Associations between total AUDIT score and lifetime substance use in both the univariable and multivariable analyses are also consistent with prior Finnish studies showing that alcohol problems are often associated with other substance use, especially cannabis use (Hakkarainen et al. 2018; Lintonen et al. 2018; Warpenius and Mäkelä 2020). Therefore, even though participants in the early midlife assessment represent a subset of the original FT12 sample, their patterns of alcohol use, as well as associations between alcohol problems and the use of other substances, are consistent with those observed in the broader Finnish population.

Similar to alcohol use and problems, reports of physical health in our sample were comparable to population statistics in Finland, where 68% of Finns report good general health (Organization for Economic Cooperation and Development). In contrast, rates of self-reported mental health conditions in our sample are higher than population averages in Finland (Organization for Economic Cooperation and Development 2019), specifically for diagnosed anxiety and depressive disorders. According to the Organization for Economic Cooperation and Development (OECD), 4% of Finns were affected by anxiety disorders, while 6% were affected by depressive disorders in 2018 (OECD 2018). In comparison, approximately 17% and 19% of our sample reported having been diagnosed with anxiety and depression within their lifetime, respectively. The higher rates of mental health conditions in our sample compared to the Finnish population could be attributed to a few factors. The OECD reports the percentage of Finns currently affected by anxiety and depression (in 2018), while the FT12 survey captures lifetime diagnosis of anxiety and depression. The latest OECD report also uses data collected prior to the COVID-19 global pandemic while the FT12 early midlife survey was collected during the public health emergency phase of the pandemic. The 2022–2023 Finnish Institute of Health and Welfare’s FinSote survey reports a deterioration of adult mental health throughout the COVID-19 global pandemic, with an increase in mental stress and use of mental health services (Suvisaari et al. 2023). Additionally, rates of self-reported mental health diagnoses in surveys such as the FT12 survey, may be heightened compared to symptom-based measures or clinical diagnoses (Boyd et al. 1982; Davies et al. 2022).

The current study is an important contribution to our knowledge about the correlates of alcohol misuse in early midlife. A sister study to the FT12 cohort, FinnTwin16, collected similar substance use, physical, and psychological health measures in early midlife (Kaidesoja et al. 2019). The birth years of the two cohorts differed by 4 to 12 years, and collection of the FinnTwin16 early midlife measures took place in early 2010s while collection of the FT12 early midlife measures occurred during the COVID-19 public health emergency. Despite the differences in birth years and the potential influence of COVID-19 on FT12 early midlife measures, alcohol use and physical and psychological health were comparable between the two samples (Lumpe et al. 2024). Similar to FT12, sex differences were reported in multiple FinnTwin16 early midlife substance use, physical, and psychological health measures (Berg et al. 2024).

Another longitudinal study conducted in the United States, Monitoring the Future (MTF), reported that 28% of early midlife participants had an alcohol use disorder (Schulenberg et al. 2016), a proportion slightly higher than the 21% of FT12 participants that reported hazardous alcohol use. MTF survey participants reported alcohol use within the past 5 years, while our early midlife survey participants reported current alcohol use only, which may contribute to the slight discrepancy in alcohol misuse prevalence rates between samples. In line with associations between AUDIT and early midlife demographic, substance use, physical, and psychological health predictors in the FT12 sample, the MTF team reported similar associations between higher alcohol misuse and worse financial situation, physical, and psychological health in early midlife (Schulenberg et al. 2016).

Reports of early midlife alcohol use and correlates are limited to samples in Finland and the United States, and future use of the FT12 early midlife data should be considered in the cultural context in which data were collected. Finland consistently scores higher than most other countries on multiple measures of the OECD Better Life Index, including educational attainment, employment rates, financial situation, and life satisfaction (Organization for Economic Cooperation and Development). In addition, the Finnish drinking culture has previously been characterized as “wet and permissive” (Mäkelä et al. 2012), and alcohol use is ingrained within societal norms. Given these population statistics and the cultural attitudes towards alcohol in Finland, the data and corresponding results of the FT12 early midlife survey may not be generalizable to high-risk or clinical populations, or to other countries where drinking norms differ.

### Future Directions

The FT12 study, including the recently added early midlife data, serves as an important resource for understanding the antecedents and consequences of health-related behaviors. Additional data collection to provide a comprehensive understanding of early midlife health is currently being planned. Similar to data collection at ages 14 and 22, the intensive subsample of FT12 will be invited to participate in an in-person assessment. This in-person assessment will include anthropometric and physiological measurements, neuropsychological testing, multiple omics, and more in-depth psychological phenotyping. The forthcoming deep phenotyping in combination with the extended longitudinal data presented here can be used to improve our understanding of the etiology of alcohol use problems and resilience as well as other health behaviors.

## Electronic Supplementary Material

Below is the link to the electronic supplementary material.

Supplementary Material 1

Supplementary Material 2

## Data Availability

Research data are not shared owing to Finnish data privacy laws.
