# Supplementary material for: Alcohol use in Early Midlife: Findings from the Age 37 Follow-Up Assessment of the FinnTwin12 Cohort
Source: Behav Genet. 2025 Feb 8;55(2):124–40. doi: 10.1007/s10519-024-10212-y (PMC11882652; doi:10.1007/s10519-024-10212-y)
Supplement: Supplementary file 1 — Supplementary Material 1 [file 10519_2024_10212_MOESM1_ESM.docx]

Supplemental Tables

**Table S1**

*Logistic Regression Analyses of Early Midlife Assessment Participation*

| Variable | OR [95% CI] |
| --- | --- |
| *Twin variables* |  |
| Sex | 2.29 [1.95, 2.72] |
| Zygosity | 0.72 [0.59, 0.87] |
| Alcohol use frequency | 1.04 [0.95, 1.15] |
| Alcohol intoxication | 1.08 [0.96, 1.22] |
| Hyperactivity/impulsivity | 0.59 [0.51, 0.70] |
| Aggression | 0.67 [0.54, 0.83] |
| Depression | 0.92 [0.76, 1.11] |
| *Parental variables* |  |
| Mother alcohol misuse | 1.05 [0.41, 1.21] |
| Father alcohol misuse | 0.95 [0.88, 1.02] |
| Mother education | 1.13 [1.07, 1.20] |
| Father education | 1.07 [1.01, 1.14] |

*Note.* All parent and twin variables (except twin alcohol use frequency and intoxication) were assessed at the baseline (age 12) assessment. Twin alcohol use frequency and intoxication were assessed at the age 14 follow-up assessment. Reference categories are male for sex and monozygotic for zygosity.
