# Supplementary material for: Alcohol use in Early Midlife: Findings from the Age 37 Follow-Up Assessment of the FinnTwin12 Cohort
Source: Behav Genet. 2025 Feb 8;55(2):124–40. doi: 10.1007/s10519-024-10212-y (PMC11882652; doi:10.1007/s10519-024-10212-y)
Supplement: Supplementary file 2 — Supplementary Material 2 [file 10519_2024_10212_MOESM2_ESM.docx]

**Supplementary Information**

Article title: Alcohol use in early midlife: Findings from the age 37 follow-up assessment of the FinnTwin12 cohort

Journal: *Behavior Genetics*

Authors: Megan E. Cooke, Erin Lumpe, Mallory Stephenson, Mia Urjansson, Fazil Aliev, Teemu Palviainen, Sarah J. Brislin, Maarit Piirtola, Jill Rabinowitz, Antti Latvala, Peter Barr, Eero Vuoksimaa, Hermine H. M. Maes, Richard Viken, Richard J. Rose, Jaakko Kaprio, Danielle M. Dick, Sari Aaltonen, Jessica E. Salvatore

Corresponding authors:

Jaakko Kaprio, Institute for Molecular Medicine Finland, University of Helsinki, Email: jaakko.kaprio@helsinki.fi

Danielle M. Dick, Department of Psychiatry, Robert Wood Johnson Medical School, Rutgers University, Email: danielle.m.dick@rutgers.edu

Sari Aaltonen, Institute for Molecular Medicine Finland, University of Helsinki, Email: sari.s.aaltonen@helsinki.fi

Jessica E. Salvatore, Department of Psychiatry, Robert Wood Johnson Medical School, Rutgers University, Email: jessica.salvatore@rutgers.edu

**FinnTwin12 Early Midlife Survey**

*Note: Items have been translated from Finnish to English.*

**Demographics**

1. Are you still living with your twin?

1. no, we lived together until the age of _____
2. yes, I am still living with my twin

2. How often do you meet him/her or keep contact with him/her in other ways? (e.g. by phone, WhatsApp, e-mail, in-person, or through social media)

1. daily or nearly every day
2. approx. once a week
3. approx. once a month
4. approx. once in a half year
5. less frequently
6. never

3. Are you living together with

1. a spouse or a partner
2. a spouse or a partner and child or children
3. a parent or both of your parents
4. alone
5. alone with your child or children
6. other (e.g., dormitory or with your siblings)

4. What schools/degrees have you completed? (you can choose several alternatives)

1. junior high school
2. vocational school or corresponding school
3. college level or corresponding level
4. senior high school
5. university of applied sciences
6. college or university

5. Are you currently

1. working (including being an entrepreneur)
2. at home (e.g., a house wife/husband, a stay-at-home mother/father)
3. a student
4. unemployed, looking for work
5. on maternity, paternity, parental or child care leave
6. retired
7. a family caregiver
8. something else, please specify_________________________

6. What is your financial situation?

1. very good
2. fairly good
3. moderate
4. fairly poor
5. very poor

7. What is your primary occupation/job title? Please be as precise as possible. For example, instead of using the term “salesperson,” you should use the term “salesperson in a grocery store.”

**^____________________________________________________________________________________________^**

**Employment**

8. How many hours do you work in a week?

1. less than 20 hours
2. 20 to 29 hours
3. 30 to 39 hours
4. 40 to 44 hours
5. 45 to 54 hours
6. 55 hours or more

9. The following questions are about how invested you are in your work and how rewarding the work is. How much do you feel:

|  | Very great extent | Great extent | Neither a little nor a lot | Little extent | Very little extent |
| --- | --- | --- | --- | --- | --- |
| You focus your abilities and resources on your work | 1 | 2 | 3 | 4 | 5 |
| You are compensated for the work you do in terms of income, employee benefits, etc. | 1 | 2 | 3 | 4 | 5 |
| You receive recognition or respect for the work you do | 1 | 2 | 3 | 4 | 5 |
| You receive personal satisfaction from the work you do | 1 | 2 | 3 | 4 | 5 |

10. How often do you have the feelings or thoughts described by the following statements?

|  | Always | Most of the time | Sometimes | Rarely | Never | Can’t Say |
| --- | --- | --- | --- | --- | --- | --- |
| I feel full of energy when I am working | 1 | 2 | 3 | 4 | 5 | 6 |
| I feel enthusiastic about my work | 1 | 2 | 3 | 4 | 5 | 6 |
| I am fully immersed in my work | 1 | 2 | 3 | 4 | 5 | 6 |

**General Health and Health History**

11. How tall are you?

_______ cm

12. What is your current weight?

_______ kg

(for women: if you are pregnant at the moment, what was your weight before the pregnancy?)

13. In your lifetime, how many times have you lost more than 5 kg of weight?

1. never
2. once
3. 2 to 4 times
4. 5 or more times

14. What do you think about your health, is it

1. very good
2. quite good
3. average
4. quite poor
5. very poor

15. How often have you had the following symptoms during the past six months?

|  | Almost daily | More than once a week | About once a week | About once a month | Less often or never |
| --- | --- | --- | --- | --- | --- |
| headache | 1 | 2 | 3 | 4 | 5 |
| stomachache | 1 | 2 | 3 | 4 | 5 |
| difficulty getting to sleep | 1 | 2 | 3 | 4 | 5 |
| waking up during sleep | 1 | 2 | 3 | 4 | 5 |
| nervousness and anxiousness | 1 | 2 | 3 | 4 | 5 |
| irritability and a fit of rage | 1 | 2 | 3 | 4 | 5 |
| lower back pain | 1 | 2 | 3 | 4 | 5 |
| neck and shoulder pain | 1 | 2 | 3 | 4 | 5 |

16. Has a physician, nurse, or other health care provider ever told you that you have now or have had sometime before any of the following diseases or injuries:

No Yes How old were you when you got the diagnosis (years)?

anxiety or panic disorder 1 2

alcohol use disorder 1 2

allergic rhinitis (hay fever) 1 2

asthma 1 2

epilepsy 1 2

attention problems/ADD/ADHD 1 2

thyroid disease 1 2

coronavirus disease 1 2

depression 1 2

migraine 1 2

ankle ligament injury 1 2

knee ligament injury or meniscus injury 1 2

post-traumatic stress disorder 1 2

sleep apnea 1 2

hypertonia or elevated blood pressure 1 2

other chronic disease 1 2

17. Do you constantly or periodically use any medication for some disease?

1 no

2 yes

18. During the past 12 months, how many whole days have you been absent from work or unable to perform your usual duties due to sickness? If you don’t remember precisely, an estimate is enough. Don’t count days missed due to pregnancy.

______ days

**COVID-Related Life Events/Stressors**

19. Overall, considering all the possible ways your life may have been impacted by the COVID-19 pandemic, how much has the pandemic impacted your day-to-day life?

1. it has not impacted my life at all -> **skip to question 21**
2. it has impacted my life a little.
3. it has moderately impacted my life.
4. it has extremely impacted my life.

20. How has the coronavirus pandemic and associated restrictions affected the following areas of your life? If some of the items are not relevant for you, please select "not applicable."

|  | No effect | Decreased | Increased | Not applicable |
| --- | --- | --- | --- | --- |
| Communication with friends, family members, and relatives | 1 | 2 | 3 | 4 |
| Loneliness | 1 | 2 | 3 | 4 |
| Feeling optimistic about the future | 1 | 2 | 3 | 4 |
| Daily physical activity | 1 | 2 | 3 | 4 |
| Income | 1 | 2 | 3 | 4 |
| Remote work | 1 | 2 | 3 | 4 |
| Time spent at school, social, or religious activities | 1 | 2 | 3 | 4 |
| Stress | 1 | 2 | 3 | 4 |
| Discord in your household | 1 | 2 | 3 | 4 |

**Physical Fitness and Activity**

21. Is your current physical fitness

1. very good
2. quite good
3. satisfactory
4. quite poor
5. very poor

22. How often do you engage in physical activity during your leisure time?

1. not at all
2. less than once a month
3. 1–2 times a month
4. once a week
5. 2–3 times a week
6. 4–5 times a week
7. nearly every day
8. several times a day

23. On average, is your physical activity during leisure time about as tiring as

1. walking
2. alternately walking and jogging
3. jogging (light run)
4. running

24. How long does one session of physical activity last, on average?

1. less than half an hour
2. half an hour to less than one hour
3. one hour to less than two hours
4. two hours or more

25. How much of your daily journey to work/study is spent walking, cycling, running and/or cross-country skiing?

1. less than 15 minutes
2. 15 minutes to less than half an hour
3. half an hour to less than one hour
4. one hour or more
5. I am presently not at work or studying

26. Which of the following descriptions best suits your work?

1. largely sedentary work, not much walking during the working day
2. sedentary or standing work involving some walking, but not much lifting or carrying
3. work involving a lot of walking, lifting, or carrying
4. heavy manual work, involving lifting or carrying heavy objects, digging, logging, etc.
5. I don't work or study

27. What is your leisure time physical activity/exercise like? (you can circle several alternatives)

1 walking/Nordic walking 14 yoga 27 padel

2 jogging 15 pilates 28 golf

3 cycling 16 stretching 29 disc golf

4 cross country skiing 17 floorball 30 slalom/snowboarding

5 swimming/aqua jogging 18 soccer 31 riding

6 roller-skating/skating 19 ice hockey 32 orienteering

7 working out in the gym 20 rink hockey 33 rowing/canoeing

8 aerobics 21 volleyball 34 martial arts

9 gymnastics 22 basketball 35 else, specify

10 circuit training 23 Finnish baseball _____________________

11 kettlebell training 24 badminton _____________________

12 spinning 25 squash _____________________

13 dance 26 tennis

28. How much time, on average, do you spend on physically demanding leisure time activities (e.g., gardening, repair work, cleaning) per day? Please, do not take into consideration work-related physical activity, commuting-related physical activity, or goal-oriented leisure time exercise.

1. less than half an hour
2. half an hour to less than one hour
3. one hour to less than two hours
4. two hours to less than three hours
5. three hours or more

29. How much time, on average, do you spend sitting in a car, reading, watching television, doing handicrafts, surfing the Internet, playing computer games, or doing other sedentary activities per day?

1. less than 1 hour
2. 1 hour to less than 2 hours
3. 2 hours to less than 4 hours
4. 4 hours or more

**Social Media Use**

30. On an average day, how much time do you spend watching TV or movies, surfing the web, playing games online, or on apps?

1. none
2. less than 1 hour
3. 1 hour to less than 3 hours
4. 3 hours to less than 6 hours
5. 6 hours or more

31. On an average day, how much time do you spend on any social media site (e.g., Facebook, Twitter, Instagram, YouTube, Pinterest, Snapchat)?

1. none
2. less than 1 hour
3. 1 hour to less than 3 hours
4. 3 hours to less than 6 hours
5. 6 hours or more

**Sleep**

32. How many hours do you usually sleep per 24 hours?

1. 6 hours or less
2. 6.5 hours
3. 7 hours
4. 7.5 hours
5. 8 hours
6. 8.5 hours
7. 9 hours
8. 9.5 hours
9. 10 hours or more

33. How do you generally sleep?

1. well
2. fairly well
3. fairly poorly
4. poorly
5. cannot say

34. Do you feel sleepiness during the day?

1. every day or nearly every day
2. 3–5 days a week
3. 1–2 days a week
4. less than once a week
5. less than once a month or never

35. Will you try to estimate to what extent you are a morning or an evening person?

1. clearly a morning person
2. somewhat a morning person
3. somewhat an evening person
4. clearly an evening person

36. How often do you suffer from insomnia?

1. every night or nearly every night
2. 3–5 nights a week
3. 1–2 nights a week
4. less than once a week
5. less than once a month or never

37. Do you snore?

1. every night or nearly every night
2. 3–5 nights a week
3. 1–2 nights a week
4. less than once a week
5. less than once a month or never
6. I don’t know

**Neighborhood Context**

38. Do you feel unsafe when walking in your neighborhood?

1. never
2. very rarely
3. quite rarely
4. quite often
5. very often

39. Are you afraid to be alone outdoors in the evenings after 22:00?

1. I do not go out alone in the evenings or I cannot tell
2. I do not go out alone in the evenings because I am afraid
3. never
4. every now and then
5. often

40. To what extent do you agree with the following statement: “I feel at home in this neighborhood”?

1. totally disagree
2. disagree
3. neither agree nor disagree
4. agree
5. totally agree
6. don’t know

**Romantic Relationships and Parenting**

41. How many relationships have you had where you have been living together with your partner?

1. none
2. one
3. two
4. three or more

42. How long has your relationship with your current partner lasted?

1. I’m not going steady with somebody -> **skip to question 47**
2. I’m going steady, I’m married, or living together with somebody. The relationship began approx. _____ years ago

43-46. Revised Dyadic Adjustment Scale (RDAS). Not included due to copyright. For the full version of this scale, see:

Busby, D. M., Christensen, C., Crane, D. R., & Larson, J. H. (1995). A revision of the Dyadic Adjustment Scale for use with distressed and nondistressed couples: Construct hierarchy and multidimensional scales. *Journal of Marital and Family Therapy*, *21*(3), 289–308. <https://doi.org/10.1111/j.1752-0606.1995.tb00163.x>

47. Are you/is your partner pregnant at the moment?

1. no
2. yes

48. Do you have children whose biological father/mother you are?

1. no
2. yes -> how many children?__________ ages?__________

49. Are there children living in your household whose biological father/mother you are not?

1. no
2. yes🡪 how many?___________ages?__________

How much do you agree or disagree with the following statements?

*Branching logic: If reported number of children = 0 (Q48 and Q49), skip the remaining items in the Parenting section*

50. I am happy in my role as parent.

1. strongly agree
2. agree
3. neither agree nor disagree
4. disagree
5. strongly disagree

51. I feel close to my child(ren).

1. strongly agree
2. agree
3. neither agree nor disagree
4. disagree
5. strongly disagree

52. A major source of stress in my life is my child(ren).

1. strongly agree
2. agree
3. neither agree nor disagree
4. disagree
5. strongly disagree

53. I feel overwhelmed by the responsibility of being a parent.

1. strongly agree
2. agree
3. neither agree nor disagree
4. disagree
5. strongly disagree

**Alcohol**

54. Have you drank alcohol before?

1. yes, I have drunk alcohol
2. no, I have never drunk alcohol -> skip to question 67

55. How often do you have a drink containing alcohol?

1. never
2. monthly or less
3. 2-4 times a month
4. 2-3 times a week
5. 4 or more times a week

56. How many drinks containing alcohol do you have on a typical day when you are drinking?

1. 1 or 2
2. 3 or 48
3. 5 or 6
4. 7 to 9
5. 10 or more

57. How often do you have six or more drinks on one occasion?

1. never
2. less than monthly
3. monthly
4. weekly
5. daily or almost daily

*One drink equals:*

*One bottle of medium strength beer or cider (33 cl)*

*One glass of wine (12 cl)*

*A small glass of strong wine (8 cl)*

*One measure of liquor (4 cl)*

58. How often during the last year have you found that you were not able to stop drinking once you had started?

1. never
2. less than monthly
3. monthly
4. weekly
5. daily or almost daily

59. How often during the last year have you failed to do what was normally expected of you because of drinking?

1. never
2. less than monthly
3. monthly
4. weekly
5. daily or almost daily

60. How often during the last year have you needed a drink in the morning to get yourself going after a heavy drinking session?

1. never
2. less than monthly
3. monthly
4. weekly
5. daily or almost daily

61. How often during the last year have you had a feeling of guilt or remorse after drinking?

1. never
2. less than monthly
3. monthly
4. weekly
5. daily or almost daily

62. How often during the last year have you been unable to remember what happened the night before because of your drinking?

1. never
2. less than monthly
3. monthly
4. weekly
5. daily or almost daily

63. Have you or someone else been injured because of your drinking?

1. no
2. yes, but not in the last year
3. yes, during the last year

64. Has a relative, friend, doctor or other health care worker been concerned about your drinking or suggested you cut down?

1. no
2. yes, but not in the last year
3. yes, during the last year

65. What is the largest amount of alcohol you have ever drunk within a 24-hour period (even if it happened just once; e.g., at Midsummer, the First of May, during cruises)? Even a rough estimate is enough.

1. ________ drinks
2. I have never drunk alcohol

*E.g. One case of beer = 24 drinks*

*A bottle of wine (0.75 l) = 6 drinks*

*A bottle of liquor (0.5 l) = 13 drinks*

*A bottle of liquor (0.7l) = 18 drinks*

66. Next, I want you to think about the time in your life **when you were drinking the most**; your heaviest drinking ever. How old were you at that time? If you are currently drinking the most now, please list your current age.

__years old

**Nicotine**

67. Over your lifetime, have you smoked more than 100 cigarettes (5 packs)?

1. no I haven’t **-> skip to question 71**
2. yes

68. Which of the following alternatives best describes your current use of cigarettes?

1. I smoke daily
2. I smoke once a week or more often, though not daily
3. I smoke less frequent than once a week
4. I have stopped or quit smoking

69. When was the last time you smoked?

1. yesterday or today
2. 2 days to less than 1 month ago
3. 1 month to less than half a year ago
4. half a year to less than a year ago
5. a year to less than 5 years ago
6. 5 years to less than 10 years ago
7. 10 years to less than 20 years ago
8. 20 years ago or earlier

**70. Think about the period in your life when you were smoking the most:**

A. How soon after you wake/woke up do/did you smoke your first cigarette?

1. within 5 minutes
2. 6 to 30 minutes
3. 31 to 60 minutes
4. after 60 minutes

B. Do/did you find it difficult to refrain from smoking in places where it is/was forbidden?

1. no
2. yes

C. Which cigarette would you hate/have hated most to give up?

1. the first one in the morning
2. any other

D. How many cigarettes per day do/did you smoke?

1. 10 or less
2. 11 to 20
3. 21 to 30
4. 31 or more

E. Do/did you smoke more frequently during the first hours after waking than the rest of the day?

1. no
2. yes

F. Do/did you smoke when you are/were so ill that you are/were in bed most of the day?

1. no
2. yes

71. Do you currently use nicotine-containing e-cigarettes?

1. daily
2. occasionally
3. no

72. Do you smoke cigars, cigarillos, or pipes?

1. never
2. once in awhile
3. regularly

73. Have you tried snuff? So far how many times altogether?

1. I have not tried
2. I have tried once
3. I have used snuff 2 to 50 times
4. I have used snuff over 50 times
5. I use snuff regularly

**Other Substance Use**

74. Have you ever tried cannabis (hash or marijuana)?

1. never
2. 1-3 times
3. 4-9 times
4. 10-19 times
5. 20 times or more

75. Have you ever used other substances to get high (thinner or other inhaled substance, amphetamine, medication on purpose to get high, or other such substances)?

1. never
2. 1-3 times
3. 4-9 times
4. 10-19 times
5. 20 times or more

**Recovery**

76. Did you used to have a problem with drugs or alcohol, but no longer do?

1. yes
2. no -> **skip to question 78**

77. Which recovery support services or treatment programs have you participated in?

**^___________________________________________________________________________________________________________________^**

**Criminal Offending**

78. Have you ever been fined for speeding?

1. no
2. yes -> how old were you when this happened for the first time? ________

79. Have you ever illegally downloaded something (e.g., music, movies, games, computer software, applications) from the Internet or from a peer-to-peer network, or have you ever used a computer for ‘hacking’?

1. no
2. yes -> how old were you when this happened for the first time? ________

80. Have you ever stolen something worth over 50 EUR from a store, house, car, or person, or have you used someone else’s credit card or bank card without their permission or knowledge?

1. no
2. yes -> how old were you when this happened for the first time? ________

81. Have you ever sold or helped sell marijuana or other drugs, such as heroin, amphetamine, or LSD?

1. no
2. yes ->how old were you when this happened for the first time? ________

82. Have you ever broken into a building (e.g., someone’s home, a shop, or a storehouse)?

1. no
2. yes ->how old were you when this happened for the first time? ________

*Branching logic: If reported “no” in all these previous questions (Q78-82), skip the remaining items in the criminal offending section.*

83. Have you ever threatened someone with violence or with a weapon when stealing something from a store, house, car, or person?

1. no
2. yes ->how old were you when this happened for the first time? ________

84. Have you ever physically assaulted another person so that (s)he was injured?

1. no
2. yes ->how old were you when this happened for the first time? ________

85. Have you ever sexually harassed or assaulted another person?

1. no
2. yes ->how old were you when this happened for the first time? ________

86. Have you ever been convicted in court (e.g., to fines, community service, probation, or prison)?

1. no
2. yes ->how old were you when this happened for the first time? ________

87. Have you ever been sentenced to prison?

1. no
2. yes->how old were you when this happened for the first time? ________

**Internalizing**

88. For each statement below, choose how often it matched your feelings and actions during the past week.

|  | Rarely or none of the time (less than 1 day) | Some or a little of the time (1-2 days) | Occasionally or a moderate amount of the time (3-4 days) | Most or all of the time (5-7 days) |
| --- | --- | --- | --- | --- |
| I felt depressed | 1 | 2 | 3 | 4 |
| I felt that everything I did was an effort | 1 | 2 | 3 | 4 |
| My sleep was restless | 1 | 2 | 3 | 4 |
| I was happy | 1 | 2 | 3 | 4 |
| I felt lonely | 1 | 2 | 3 | 4 |
| I enjoyed life | 1 | 2 | 3 | 4 |
| I felt sad | 1 | 2 | 3 | 4 |
| I could not get “going.” | 1 | 2 | 3 | 4 |

**Lifetime Traumatic Events**

89. Next, a number of events that can happen or serious/exceptional events in life are listed. Has any of the following events happened to you in your lifetime?

Divorce or separation

1. no
2. yes

The death of someone important and close to you

1. no
2. yes

Disease or injury causing over three weeks of work disability

1. no
2. yes

A serious traffic accident or other serious accident

1. no
2. yes

Fire or catastrophe

1. no
2. yes

Hit or kicked hard enough to get injured

1. no
2. yes

Someone forced/tried to force sexual contact with you

1. no
2. yes

A violent crime where a gun, a knife, or some other weapon was used

1. no
2. yes

Some other very traumatic event: __________________

**Relationships**

90. Do you have any close family members or other relatives (sisters, parents, cousins, etc.) whom you feel at ease with, can talk to about private matters, and can call on for help? How many close family members/relatives do you have?

1. none
2. 1 or 2
3. 3 to 5
4. 6 to 9
5. 10 or more

**Friends**

91. Close friends are people whom you feel at ease with, can talk to about private matters and can call on for help. How many close friends do you have?

1. none
2. 1 or 2 friends
3. 3 to 5 friends
4. 6 to 9 friends
5. 10 or more friends

**Now I’d like to ask a few questions about your close friends’ behavior in the last 6 months.**

92. How many of your close friends smoked cigarettes or used other tobacco products (e-cigarettes, cigars, cigarillos, snuff, etc.) regularly (more days than not).

1. none of them
2. a few of them
3. most of them
4. all of them

93. How many of your close friends got drunk regularly (once a week or more)?

1. none of them
2. a few of them
3. most of them
4. all of them

94. How many of your close friends used marijuana or other substances to get high regularly (once a week or more)?

1. none of them
2. a few of them
3. most of them
4. all of them

95. Have you ever experienced physical, psychological, or sexual violence or abuse in any past intimate relationships?

1. no
2. yes

96. Does the violence you experienced still affect your health, well-being, or life management?

1. no
2. yes

97. Is there any physical, psychological, or sexual violence or abuse in your current intimate or close relationships?

1. no
2. yes

**If responded “yes” to questions 95, 96, or 97:**

98. Who has been violent towards you?

1. spouse or partner
2. other family member
3. close relative or friend

99. When he/she has been violent towards you, how often were they under the influence of alcohol or drugs?

1. rarely or none of the time
2. some or a little of the time
3. occasionally
4. most or all of the time

**Life Satisfaction**

100. Below you’ll find five statements about life. You may agree or disagree with each of them. For each item, please circle how much it describes your situation using numbers 1 to 7.

|  | Strongly disagree | Disagree | Slightly disagree | Neither agree nor disagree | Slightly agree | Agree | Strongly agree |
| --- | --- | --- | --- | --- | --- | --- | --- |
| In most ways, my life is close to ideal | 1 | 2 | 3 | 4 | 5 | 6 | 7 |
| The conditions of my life are excellent | 1 | 2 | 3 | 4 | 5 | 6 | 7 |
| I am satisfied with my life | 1 | 2 | 3 | 4 | 5 | 6 | 7 |
| So far, I have gotten the important things I want in life | 1 | 2 | 3 | 4 | 5 | 6 | 7 |
| If I could live my life over, I would change almost nothing | 1 | 2 | 3 | 4 | 5 | 6 | 7 |

101. Finally, we ask you to measure around your waist with the measuring tape we sent you along with the invitation letter. Please stand up straight when measuring. Measure the slimmest point of your waist. If you have difficulties in finding it, measure as shown at the picture, the circumference situated in the middle of the lowest part of the ribs (A) and the upper part of the hip bone (B).

My waist measurement is _______ cm

*
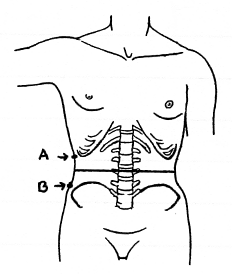
*
